# Supplementary material for: From sequence to enzyme mechanism using multi-label machine learning
Source: BMC Bioinformatics. 2014 May 19;15:150. doi: 10.1186/1471-2105-15-150 (PMC4229970; doi:10.1186/1471-2105-15-150)
Supplement: Additional file 2 — Java code of ml2db. Additional file ml2db_code.tar.gz contains the Java source code to run the multi-label machine learning experiments and save the results to database. The code’s Javadoc is included. [file 1471-2105-15-150-S2.zip › additional file 2/ml2db/ml2db/doc/mulan/classifier/neural/ThresholdFunction.html]

ThresholdFunction


JavaScript is disabled on your browser.


- Overview
- Package
- Class
- Use
- Tree
- Deprecated
- Index
- Help

- Prev Class
- Next Class

- Frames
- No Frames

- All Classes

- Summary:
- Nested |
- Field |
- Constr |
- Method

- Detail:
- Field |
- Constr |
- Method


mulan.classifier.neural

## Class ThresholdFunction

- java.lang.Object
- - mulan.classifier.neural.ThresholdFunction

- All Implemented Interfaces:
  :   java.io.Serializable

  ---

    

  ```
  public class ThresholdFunction
  extends java.lang.Object
  implements java.io.Serializable
  ```

  Implementation of a threshold function.

  Version:
  :   2012.02.27

  Author:
  :   Jozef Vilcek

  See Also:
  :   Serialized Form

- - ### Constructor Summary

    Constructors

    Constructor and Description

    `ThresholdFunction(double[][] idealLabels,
    double[][] modelOutLabels)`

    Creates a new instance of `ThresholdFunction` and
    builds the function based on input parameters.
  - ### Method Summary

    Methods

    | Modifier and Type | Method and Description |
    | --- | --- |
    | `void` | `build(double[][] idealLabels, double[][] modelOutLabels)` Build a threshold function for based on input data. |
    | `double` | `computeThreshold(double[] labelsConfidences)` Computes a threshold value, based on learned parameters, for given labels confidences. |

    - ### Methods inherited from class java.lang.Object

      `equals, getClass, hashCode, notify, notifyAll, toString, wait, wait, wait`

- - ### Constructor Detail


    - #### ThresholdFunction

      ```
      public ThresholdFunction(double[][] idealLabels,
                       double[][] modelOutLabels)
      ```

      Creates a new instance of `ThresholdFunction` and
      builds the function based on input parameters.

      Parameters:
      :   `idealLabels` - the ideal output for each input patterns, which a model should output
      :   `modelOutLabels` - the real output of a model for each input pattern

      Throws:
      :   `java.lang.IllegalArgumentException` - if dimensions of input arrays does not match

      See Also:
      :   `build(double[][], double[][])`
  - ### Method Detail


    - #### computeThreshold

      ```
      public double computeThreshold(double[] labelsConfidences)
      ```

      Computes a threshold value, based on learned parameters, for given labels confidences.

      Parameters:
      :   `labelsConfidences` - the labels confidences

      Returns:
      :   the threshold value

      Throws:
      :   `java.lang.IllegalArgumentException` - if the dimension of labels confidences does not match
          the dimension of learned parameters of threshold function.


    - #### build

      ```
      public void build(double[][] idealLabels,
               double[][] modelOutLabels)
      ```

      Build a threshold function for based on input data.
      The threshold function is build for a particular model.

      Parameters:
      :   `idealLabels` - the ideal output for each input patterns, which a model should output.
          First index is expected to be number of examples and second is the label index.
      :   `modelOutLabels` - the real output of a model for each input pattern.
          First index is expected to be number of examples and second is the label index.

      Throws:
      :   `java.lang.IllegalArgumentException` - if dimensions of input arrays does not match


- Overview
- Package
- Class
- Use
- Tree
- Deprecated
- Index
- Help

- Prev Class
- Next Class

- Frames
- No Frames

- All Classes

- Summary:
- Nested |
- Field |
- Constr |
- Method

- Detail:
- Field |
- Constr |
- Method
